# Acute internal carotid artery occlusion due to dissection of the paraclinoid segment: Diagnostic usefulness of angiographic findings during stent retriever deployment

**DOI:** 10.1016/j.radcr.2022.10.017

**Published:** 2022-10-31

**Authors:** Isao Sasaki, Taichiro Imahori, Tatsuya Yano, Kana Onobuchi, Masanori Gomi, Junko Kuroda, Norikata Kobayashi, Kimitoshi Sato, Yoji Niwa, Koichi Iwasaki, Hiroshi Hasegawa

**Affiliations:** a Department of Neurosurgery, Ainomiyako Neurosurgery Hospital, Osaka, Japan; b Department of Neurosurgery, Kobe University Graduate School of Medicine, 7-5-2, Kusunoki-cho, Chuo-ku, Kobe City, Hyogo 650-0017, Japan

**Keywords:** Acute ischemic stroke, Dissection, Large vessel occlusion, Endovascular treatment, Mechanical thrombectomy, Stent retriever

## Abstract

Intracranial artery dissection is an uncommon but possible cause of ischemic stroke, and is usually diagnosed based on imaging findings such as mural hematoma and dissection flap. However, it is challenging to recognize the underlying dissection in cases of acute large vessel occlusion. In this report, we present a case of acute internal carotid artery occlusion, in which the underlying dissection of the paraclinoid segment was found during the thrombectomy procedure. Two thrombectomy procedures failed to recanalize the acute internal carotid artery occlusion without removing any clot. Angiography performed during a Trevo stent retriever deployment in the first pass showed obscure contrast defects in the stent strut with temporary flow restoration. In the next pass, the appearance of the contrast defects changed and a parallel linear contrast appeared on the outside of the vessel wall. These angiographic findings were identified as mural hematoma and dissection flap, indicating dissection of the paraclinoid as the cause of the occlusion. During antiplatelet loading and preparation of a dedicated intracranial stent, the Trevo stent retriever was left deployed again at the occlusion site to maintain the blood flow. After permanent stenting with an Enterprise stent, angiography revealed complete recanalization. The patient recovered fully after the procedure. In the present case, stent retriever deployment revealed the hallmarks of dissection on angiography, such as mural hematoma, dissection flap, and temporal morphological changes, by restoring the blood flow temporarily. Such angiographic findings can provide useful information on the occlusion characteristics and real-time feedback for optimal treatment strategy.

## Introduction

Spontaneous intracranial artery dissection is an uncommon but known cause of ischemic stroke [1,2]. Intracranial artery dissection is usually diagnosed based on magnetic resonance (MRI) or other imaging findings, such as mural hematoma, false lumen with dissection flap, progressive irregular stenosis or occlusion, and fusiform enlargement of the external caliber of the vessel [2], [3], [4], [5], [6]. However, when blood flow at the dissected artery is completely stopped, it is difficult to identify the underlying dissection at the occlusion site on conventional angiography alone [2].

This diagnostic difficulty may pose a challenge in case of mechanical thrombectomy for patients with acute ischemic stroke due to acute large vessel occlusion, because there is limited time to obtain sufficient information from detailed images, including various MRI sequences [7], [8], [9]. Furthermore, when dissection is the underlying cause of the occlusion, frequent thrombectomy procedures in such pathologic vessels could even exacerbate the degree of the dissection. Therefore, the information obtained in the interventional radiology suite can be valuable for the endovascular treatment of acute ischemic patients.

In this article, we present a case of acute intracranial internal carotid artery (ICA) occlusion wherein the underlying dissection of the paraclinoid segment was found during the thrombectomy procedure. This case highlights the diagnostic utility of angiographic findings during stent retriever deployment to identify the cause of the occlusion.

## Case presentation

**Fig. 1:**
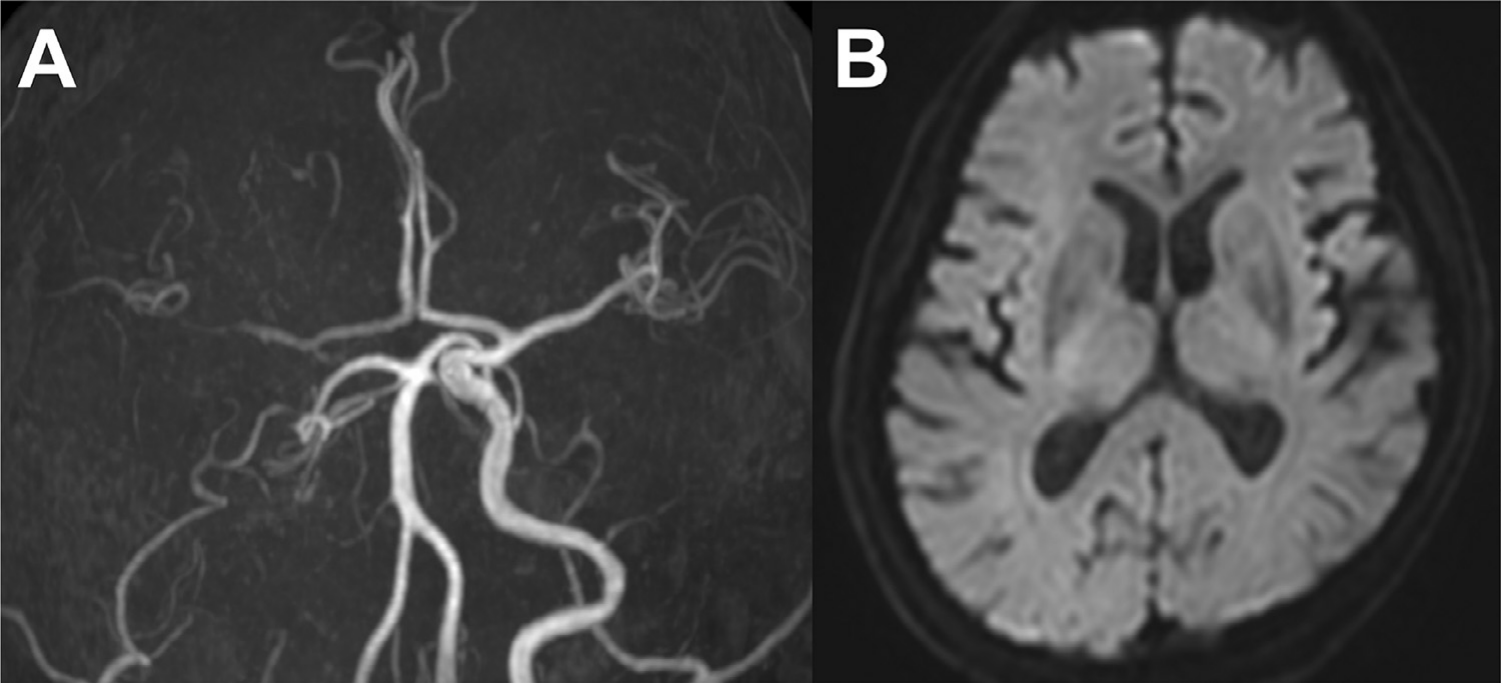
Pre-procedural imaging. (A) Magnetic resonance angiography showing occlusion of the right internal carotid artery. The right middle cerebral artery is weakly visible with collateral flow through the anterior communicating artery. (B) Diffusion-weighted imaging showing no apparent acute ischemic changes.

A man in his early 60s with a chief complaint of headache was admitted to our hospital 60 min after onset. Neurological examination showed mild dysarthria, left hemiparesis, and left hemispatial neglect. The initial National Institutes of Health Stroke Scale score was 9. The patient did not complain of any pain and denied any trauma to the head or neck. Additionally, no signs suggesting connective tissue diseases were noted. MRI revealed occlusion of the right ICA without apparent acute ischemic changes (Fig. 1A and B). The right middle cerebral artery was depicted weakly with collateral flow through the anterior communicating artery. Intravenous tissue plasminogen activator was initiated, but the patient's symptoms did not improve. Subsequently, we decided to perform endovascular revascularization therapy. Written informed consent was obtained from the patient's family member before the procedure.

**Fig. 2:**
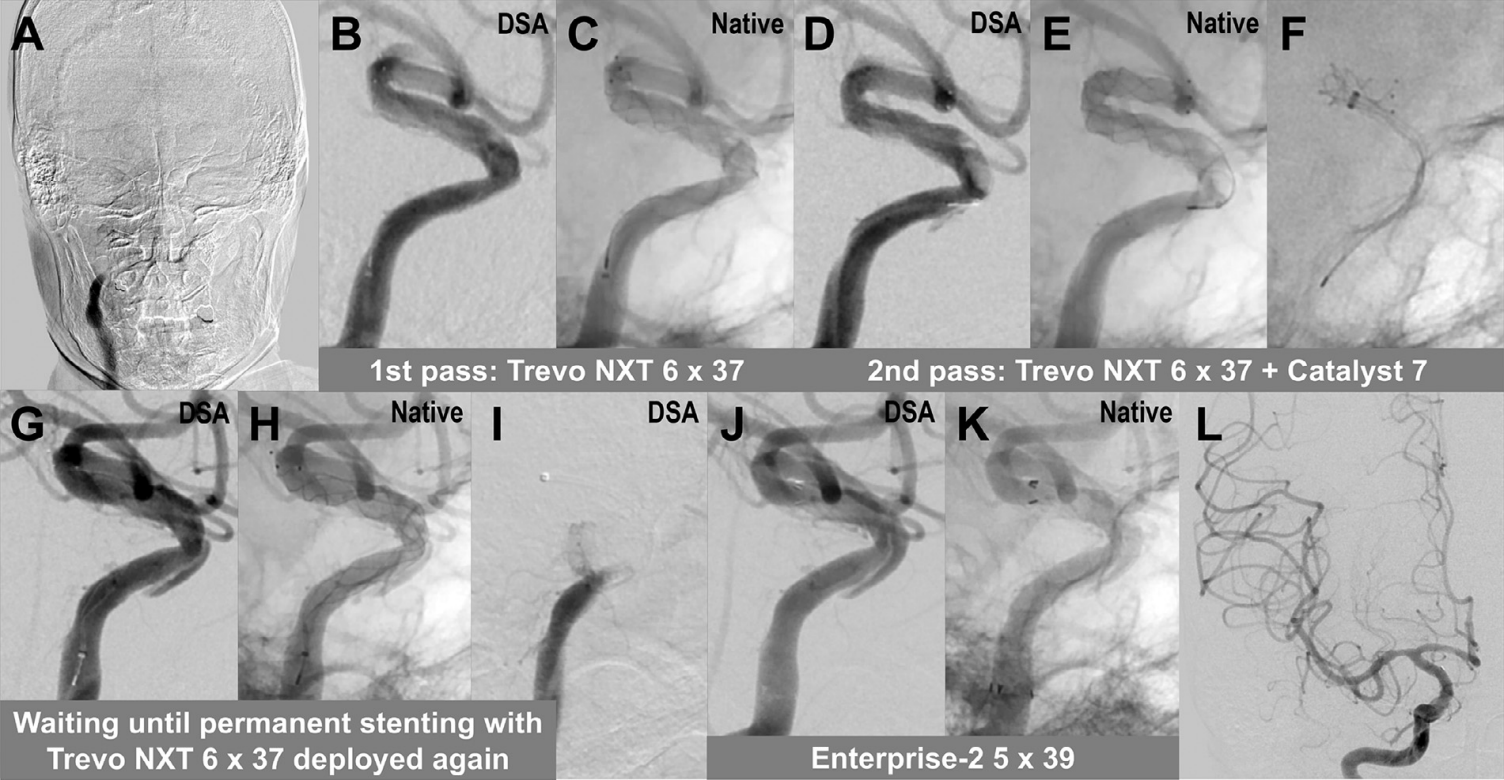
Intra-procedural imaging. (A) Initial angiography showing occlusion of the right internal carotid artery. (B and C) In the first pass using a Trevo NXT 6 × 37 mm stent retriever, digital subtraction angiography (DSA) and native angiography in the lateral projection show blood flow restoration with obscure contrast defects in the stent. (D-F) In the second pass using the Trevo stent retriever and a Catalyst 7 aspiration catheter, angiography shows blood flow restoration with changes in the morphology of the contrast defects as well as a parallel linear contrast on the outside of the vessel wall. These angiographic findings, which were identified as mural hematoma and false lumen with dissection flap, indicated the presence of dissection on the paraclinoid segment. (G and H) During antiplatelet loading and preparation of a dedicated intracranial stent, the Trevo stent retriever was left deployed to maintain the blood flow. (I) After the Trevo was resheathed and removed from the microcatheter, the artery became occluded again. (J and K) An Enterprise-2 5 × 39 mm stent was deployed as permanent stenting. (L) Final angiography revealing complete recanalization.

The endovascular procedure was performed via femoral access under local anesthesia. Heparin was not administered because of the ongoing administration of tissue plasminogen activator. A 9 French balloon guiding catheter was placed in the right cervical ICA. Initial angiography revealed occlusion of the right ICA (Fig. 2A). After the occlusion was crossed with a microcatheter and a microguidewire, a Trevo NXT 6 × 37 mm stent retriever (Stryker, Kalamazoo, MI, USA) was deployed from the proximal middle cerebral artery to the cavernous ICA. Angiography performed during stent retriever deployment showed obscure contrast defects in the stent strut with immediate flow restoration (Fig. 2B and C). However, the stent retriever failed to recanalize the occlusion and did not remove any pieces of the clot. For the second pass, we applied the combined thrombectomy technique using the Trevo stent retriever and a Catalyst 7 aspiration catheter (Stryker) (Fig. 2D-F). Angiography during stent deployment again showed immediate flow restoration with changes in the appearance of the contrast defects as well as a newly-emerged parallel linear contrast on the outside of the vessel wall. This pass also failed to recanalize the occlusion and did not remove any clots. We then recognized these angiographic findings as mural hematoma and dissection flap, which indicated that dissection of the paraclinoid segment was the cause of the occlusion. During antiplatelet loading (200 mg aspirin and 300 mg clopidogrel) and preparation of a dedicated intracranial stent, the Trevo stent retriever was left deployed to maintain the blood flow (Fig. 2G and H). Cone-beam computed tomography was performed to confirm that there was no intracranial hemorrhage. The lesion became occluded again after the Trevo was resheathed and removed from the microcatheter during the exchange method to keep the microcatheter in the dissected vessel (Fig. 2I). After permanent stenting with an Enterprise-2 stent (Cerenovus, Irvine, CA), angiography revealed thrombolysis in cerebral infarction 3 complete recanalization (Fig. 2J-L). The time from femoral access to recanalization (the third deployment of the Trevo) was 44 minutes, and the time from stroke onset to recanalization was 159 min.

**Fig. 3:**
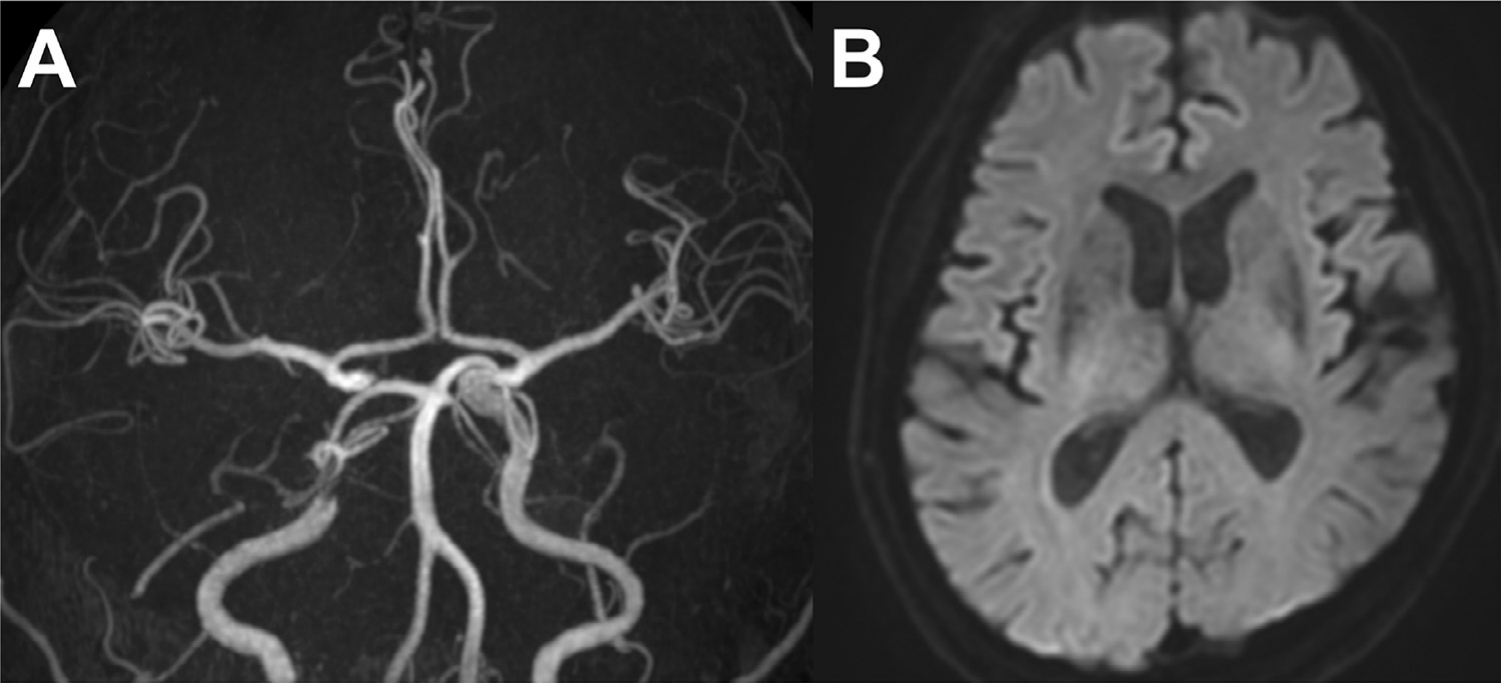
Post-procedural imaging. (A) Magnetic resonance angiography performed on the day after the procedure, showing successful revascularization. (B) Diffusion-weighted imaging showing no apparent acute ischemic changes.

**Fig. 4:**
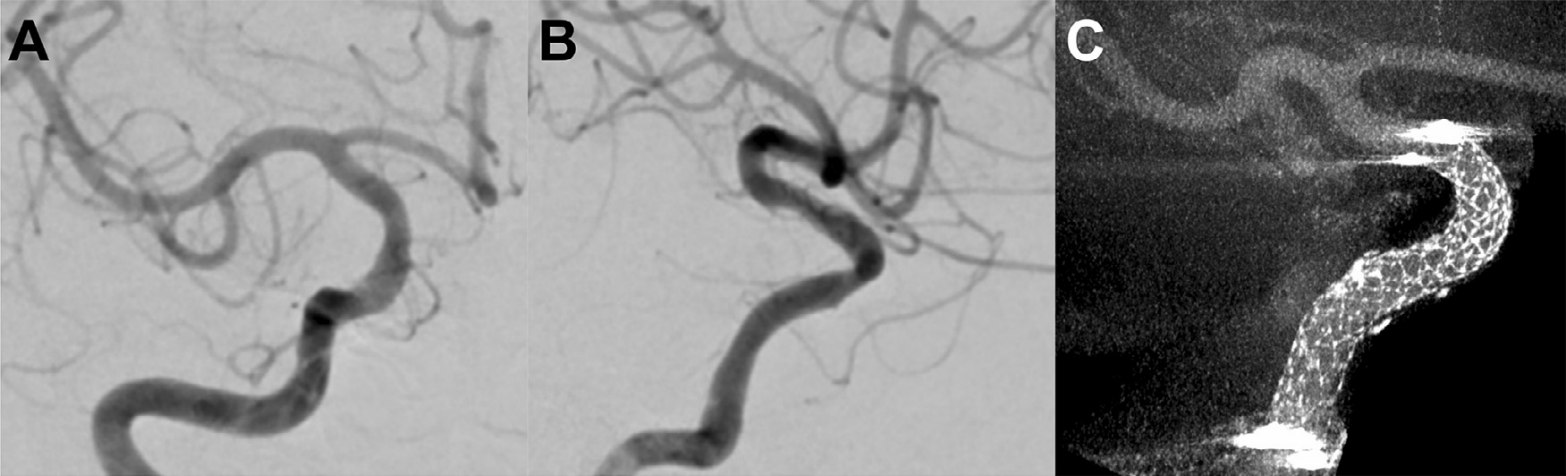
Follow-up imaging. (A and B) Six months after the procedure, follow-up angiography in the frontal and lateral projections showing resolution of dissection with minimal stenosis. (C) Cone-beam computed tomography imaging showing sufficient stent-wall apposition.

Administration of argatroban was started immediately after the procedure, and was continued for 48 h alongside dural oral antiplatelet therapy. Computed tomography performed after the procedure did not reveal any intracranial hemorrhage. The day after the procedure, MRI revealed successful revascularization without any infarction (Fig. 3A and B). The patient fully recovered and was discharged home 5 days postoperatively. At 6 months postoperatively, follow-up angiography confirmed the resolution of the dissection with minimal stenosis (Fig. 4A-C).

## Discussion

We reported a case of acute intracranial ICA occlusion, in which the underlying dissection of the paraclinoid segment was found during the thrombectomy procedure. In the present case, angiography during stent retriever deployment revealed the hallmarks of dissection, such as the mural hematoma, dissection flap, and temporal morphological changes.

This case of acute ICA occlusion was found during the thrombectomy procedure to be caused by dissection of the paraclinoid segment. To date, there exist a rather limited number of case studies on acute large vessel occlusions due to intracranial arterial dissection [6,10]. Labeyrie et al. reported that intracranial artery dissection was the underlying cause of occlusion in 13 out of 391 patients (3%) that were considered candidates for mechanical thrombectomy [6]. Among those 13 cases with intracranial dissection, ICA was involved in only 3 cases with terminal ICA dissection. In our experience of approximately 280 mechanical thrombectomy procedures over the past 6 years, intracranial ICA dissection was identified in only this single case (0.4%) [11], [12], [13], [14]. The present case demonstrated that acute cerebral artery occlusion, although rare, can occur with a dissection more proximal to the supraclinoid lesion.

In the present case, the etiology was considered spontaneous dissection without any specific reasons. However, iatrogenic dissection cannot be ruled out because it is well known that vascular injury can occur with thrombectomy procedures [15,16]. In addition, there is a high risk that the dissection is exacerbated by multiple procedures, as observed in this case. It is therefore necessary to pay close attention to the information obtained in the interventional radiology suite.

**Fig. 5:**
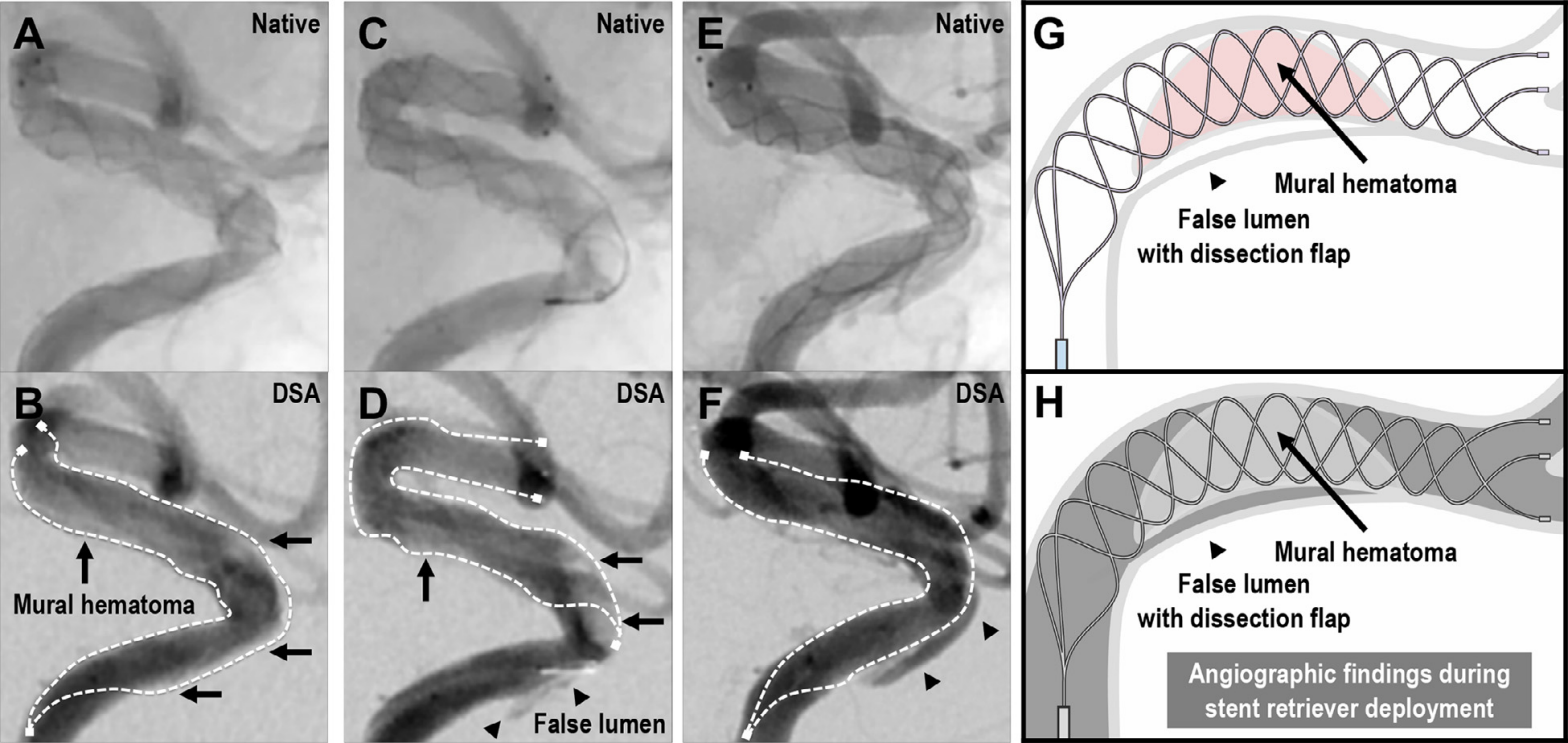
Angiographic findings during stent retriever deployment. (A and B) Native angiography and digital subtraction angiography (DSA) in the lateral projection in the first pass. (C and D) Angiography in the second pass.(E and F) Angiography during stent deployment until permanent stenting. (G and H) Scheme of a stent retriever deployed at the dissection site and corresponding angiographic image. With each deployment of the stent retriever, the caliber of the artery expanded, allowing blood flow restoration. Angiographically, mural hematoma can be observed as a contrast defect in the stent (black arrow). False lumen with dissection flap is visible as a linear contrast parallel to the vessel outside of the stent (black arrowhead). Morphological changes during the procedures are further hallmarks of dissection.

This case highlights the diagnostic utility of angiographic findings during stent retriever deployment to recognize the underlying dissection. Our previous studies have shown that angiographic images during stent retriever deployment can provide useful information [17], [18], [19], [20]. Even if recanalization is not achieved after the procedure, the occlusion characteristics can be evaluated based on the temporary restoration of blood flow during stent retriever deployment (Fig. 5A-H). Angiographically, mural hematoma and false lumen with dissection flap can be recognized as contrast defects in the stent and a linear contrast parallel to the vessel outside of the stent, respectively. Morphological changes during the procedures are also hallmarks of dissection. Such angiographical findings can provide useful information on the occlusion characteristics and real-time feedback for optimal treatment strategy. Further research is needed to generalize and utilize the information from these angiographic findings.

## Patient consent

Informed consent has been obtained from the patient's family member for publication of the case report and accompanying images.
